# Complementary pharmacological and toxicological characterization data on the pharmacological profile of *N*-(2,6-dichlorophenyl)-2-(4-methyl-1-piperidinyl) acetamide

**DOI:** 10.1016/j.dib.2016.07.019

**Published:** 2016-07-16

**Authors:** Myrna Déciga-Campos, Gabriel Navarrete-Vázquez, Francisco Javier López-Muñoz, Tadeusz Librowski, Amanda Sánchez-Recillas, Victor Yañez-Pérez, Rolffy Ortiz-Andrade

**Affiliations:** a Sección de Estudios de Posgrado e Investigación de la Escuela Superior de Medicina, Instituto Politécnico Nacional, Ciudad de México, México; b Facultad de Farmacia de la Universidad Autónoma del Estado de Morelos, Cuernavaca, Morelos, México; c Departamento de Farmacobiología, Cinvestav, Sede Sur, Ciudad de México, México; d Departament of Pharmacodynamics, Medical College, Jagiellonian University, Medyczna 9, 30-688 Kraków, Poland; e Laboratorio de Farmacología, Facultad de Química, Universidad Autónoma de Mérida, Yucatán, México

**Keywords:** LIA, *N*-(2,6-dichlorophenyl)-2-(4-methyl-1-piperidinyl)acetamide, MNPCE, micronucleated polychromatic erythrocytes, PCE, polychromatic erythrocytes, NCE, normochromatic erythrocytes, IC_50_, half maximal inhibitory concentration, LD_50_, half lethal dose, LC_50_, half lethal concentration, CYP-P450, cytochrome P-450, HR, heart rate, SBP, systolic blood pressure, DBP, diastolic blood pressure, *N*-(2,6-dichlorophenyl)-2-(4-methyl-1-piperidinyl)acetamide, Toxicity, Lidocaine

## Abstract

This text presents complementary data corresponding to pharmacological and toxicological characterization of *N*-(2,6-dichlorophenyl)-2-(4-methyl-1-piperidinyl)acetamide (LIA) compound. These data support our research article entitled “Pharmacological profile of *N*-(2,6-dichlorophenyl)-2-(4-methyl-1-piperidinyl)acetamide, a novel analog of lidocaine” Déciga-Campos M., Navarrete-Vázquez G., López-Muñoz F.J., Librowski T., Sánchez-Recillas A., Yañez-Pérez V., Ortiz-Andrade R. (2016) [1]. Toxicity was predicted through the ACD/ToxSuite software and evaluated *in vivo* using brine shrimp larvae (*Artemia salina* L.) and mice. Also, we used the micronucleus assay to determine genotoxicity. We used the platform admetSAR to predict absorption properties of LIA and lidocaine.

**Specifications Table**

**Table:** 

Subject area	Pharmacology, Toxicology
More specific subject area	Acute toxicity, cardiovascular response
Type of data	Table, figure
How data was acquired	AdmetSAR and ACD/ToxSuite software
	Pharmacological and toxicological assays *in vivo*
Data format	Analyzed
Experimental factors	Drugs were administered to mice by the oral route and toxicity parameters were observed at 14 days. Heart rate, systolic and diastolic blood pressure were evaluated in rats
Experimental features	Cardiovascular responses were measured with the Panlab Non-Invasive Blood Pressure System for Rodents and Dogs (Harvard Apparatus)
	Platform admetSAR, ACD/ToxSuite software, GraphPad Prism 5.0
Data source location	Mexico City, Mexico
Data accessibility	Data found in this article

**Value of the data**

• These data are useful to demonstrate that LIA, a new analog of lidocaine, is not toxic in Brine Ship and mice.
• LIA is not genotoxic compound in mice.
• This drug, in contrast to lidocaine, does not modify cardiovascular responses.
• LIA absorption (blood brain barrier permeability and intestinal absorption) predicted is 30% similar to that observed with lidocaine.

## 1 Data

Table 1 shows theoretical predictive values of toxicity of lidocaine and LIA determined by the ACD/ToxSuite software.

**Fig. 1:**
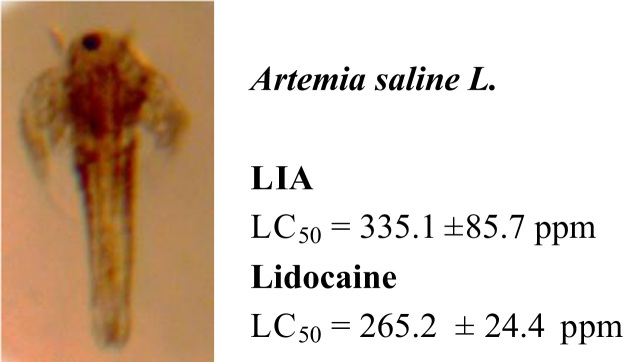
Toxicity of LIA and lidocaine in Brine Ship (*Artemia saline* L.).

**Fig. 2:**
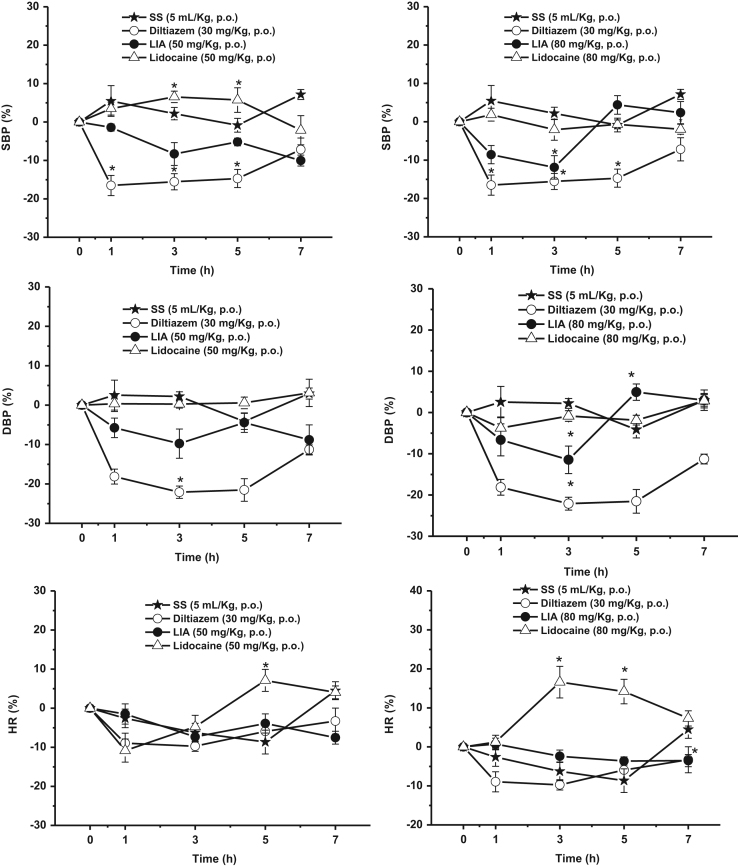
Maximal decrease in (A) systolic blood pressure, (B) diastolic blood pressure and (C) heart rate elicited by oral administration of 50 and 80 mg/kg of LIA and lidocaine in conscious rats. Results are expressed as the mean and S.E.M, *n*=6 rats per group, *p<0.05 compared with lidocaine, by repeated measures one-way analysis of variance.

Fig. 1 depicts preliminary data of toxicity assayed in Brine Ship (*Artemia saline* L.). Table 2 describes acute toxicity in two phases by the Lorke method in mice. Table 3 depicts acute genotoxicity in mice. Fig. 2 shows the cardiovascular effects of LIA and lidocaine in rats.

Table 4 depicts predictive absorption values of LIA and lidocaine.

## 2 Experimental design, materials and methods

### 2.1 Acute toxicity study

#### 2.1.1 Acute toxicity parameters of LIA and lidocaine were computed with the ACD/ToxSuite software (v. 2.95) (Table 1)

#### 2.1.2 Artemia saline lethality test

LIA and lidocaine were evaluated for lethality in brine shrimp larvae (*Artemia saina* L.) according to the procedure described previously [2]. Each concentration of either LIA or lidocaine (100, 200, 400, 800 and 1000 ppm) was assayed in triplicate. The surviving shrimp were counted after 24 h and the percentage of deaths was determined by the computation of half lethal concentration 50 (LC_50_) (Fig. 1).

#### 2.1.3 Toxicity in mice by Lorke method

Experiments were performed on ICR male mice by the Lorke method [3]. Doses were selected according this method. In both phases, mice were observed daily for 14 days for mortality, toxic effects and behavioral changes. Restlessness, respiratory distress, seizures, diarrhea, motor activity, posture and reflexes were qualitatively determined. Body weight was also monitored. The internal organs (including the stomach, heart, lung, liver, and kidneys) were removed at the end of experiment and visually examined for lesions. Neither LIA nor lidocaine produced visible macroscopically damage.

#### 2.1.4 Determination of genotoxicity by the bone marrow micronucleus assay

This test was carried out following standard protocols [4], [5]. Briefly, ICR male mice (25–30 g) were injected with cyclophosphamide (40 mg/kg, i.p.), as positive control, LIA (100 mg/kg, i.p.), lidocaine (100 mg/kg, i.p.) or vehicle (saline solution, 0.9%, i.p). Animals were sacrificed 24 h later and the bone marrow from both femurs was flushed out using 2 mL of saline and centrifuged for 5 min at 3000 rpm. The supernatant was discarded, and the pellet was re-suspended in 0.3 mL of saline. Of this smears were made on glass slides. The slides were fixed with methanol and stained with 10% Wright–Giemsa stain. Cells were blindly scored using a light microscope at 100 X magnification. For the analysis of micronucleated cells, 1000 polychromatic erythrocytes (PCE) per animal were scored. In order to assess the cytotoxic effects of compounds, the ratio of PCE to normochromatic erythrocytes (NCE) was determined in 1000 erythrocytes [4], [5]. The results are presented as the mean number of micronucleated polychromatic erythrocytes (MNPCE) or the ratio PCE:NCE in individual mice (two smears per animal)±SEM for five animals per group (Table 3).

### 2.2 Cardiovascular response in rats

The hypotensive activity was determined using a standard protocol of Avila-Villarreal et al. [6]. This study was conducted in normotensive rats. Animals were allotted into four groups (of six animals) as follows: control rats (SS, group 1), and positive control (diltiazem, group 2), LIA (group 3) and lidocaine (group 4). Treated groups were administered with diltiazem (calcium channel blocker; 30 mg/kg, p.o.), LIA (50 and 80 mg/kg, p.o.) and lidocaine (50 and 80 mg/kg, p.o.). Systolic and diastolic blood pressure as well as heart rate were recorded before and after treatment at 0, 1, 3, 5 and 7 h by the tail cuff method using a Panlab non-invasive blood pressure system for rodents and dogs (Harvard Apparatus). Percent of reduction in heart rate (HR), systolic blood pressure (SBP) or diastolic blood pressure (DBP) were calculated using diltiazem as control (Fig. 2).

### 2.3 Pharmacokinetic parameters

We used the platform admetSAR [7] to calculate human intestinal absorption, blood–brain barrier penetration, Caco-2 permeability, renal organic cation transporter, P-glycoprotein substrate and inhibitor. These properties play key roles in the discovery/development of drugs (Table 4). The software predicted high values of blood–brain barrier permeability and intestinal absorption for LIA. However, the software predicted a moderate permeability for LIA in Caco-2 cells. Furthermore, the software predicted that LIA is a P-glycoprotein substrate but not an inhibitor, indicating that this compound could have easily cleared from cells. The software also predicted that LIA is not an inhibitor of renal organic cation transporter.

## Figures and Tables

**Table 1:** Toxicity profiles predicted for LIA and lidocaine by the ACD/ToxSuite software.

**Compound**	LD_50_ (mg/kg)				Probability of inhibition			
					(IC_50_<10 μM)			
	Mouse		Rat		CYP-450	Isoform		hERG
	i.p.	p.o.	i.p.	p.o.	3A4	2D6	1A2	
**LIA**	290	460	290	1900	0.01	0.16	0.01	0.69
**Lidocaine**	130	440	130	700	0.01	0.14	0.05	0.05

**Table 2:** Acute toxicity in mice by the Lorke Method.

**Compound**	**Doses (mg/kg, i.p.)**	**Dead mice**
Lidocaine⁎		
Phase I	10	0/3
	100	0/3
	1000^c^	3/3
Phase II	140	1/3
	225^c^	1/3
	370^c^	3/3
	600^c^	3/3
LIA⁎⁎		
Phase I	10	0/3
	100	0/3
	1000	3/3
Phase II	140	0/3
	225	0/3
	370	0/3
	600	3/3

⁎ LD_50_ for lidocaine=570 mg/kg, i.p.

⁎⁎ LD_50_ for LIA=800 mg/kg, i.p.

c Mice presented seizures before death.

**Table 3:** Acute genotoxic effects of LIA and lidocaine in the bone marrow micronucleus test in mice.

**Treatment**	**Dose (mg/kg, i.p.)**	**MNPCE per 1000 PCE**	**PCE:NCE**
**Vehicle**	—	0	22.8±1.3#
**LIA**	100	5.8±1.9⁎^,^#	20.7±1.6#
**Lidocaine**	100	4.6±2.1⁎^,^#	19.5±1.4#
**Cyclophosphamide**	40	82.6±1.8#	14±0.3*

MNPCE: Micronucleated polychromatic erythrocytes.

PCE: Polychromatic erythrocytes.

NCE: Normochromatic erythrocytes.

⁎ Significantly different from cyclophosphamide (*p*<0.05).

# Significantly different from vehicle (*p*<0.05).

**Table 4:** Predictive pharmacokinetic values calculated with admetSAR for the most active compounds.

**Model**	**Probability of property of compounds**	
	**LIA**	**Lidocaine**
Blood–brain barrier^a^	(+) 0.9541	(+) 0.9688
Human intestinal absorption^b^	(+) 0.9565	(+) 1.000
Caco-2 permeability^c^	(+) 0.6075	(−) 0.6318
P-glycoprotein substrate^d^	(Y) 0.8415	(Y) 0.5806
P-glycoprotein inhibitor^e^	(N) 0.7956	(N) 0.8910
Renal organic cation transporter^e^	(N) 0.5159	(N) 0.5776

a (+) High BBB permeability; (−) moderate-poor BBB permeability.

b (+) More than 30%; (−) less than 30%.

c (+) High Caco-2 permeability; (−) moderate-poor Caco-2 permeability.

d (Y) Substrate, (N) Non-substrate.

e (Y) Inhibitor, (N) Non-inhibitor.
