# Supplementary material for: Complementary pharmacological and toxicological characterization data on the pharmacological profile of N-(2,6-dichlorophenyl)-2-(4-methyl-1-piperidinyl) acetamide
Source: Data Brief. 2016 Jul 16;8:1007–12. doi: 10.1016/j.dib.2016.07.019 (PMC5156471; doi:10.1016/j.dib.2016.07.019)
Supplement: Supplementary file 1 — Supplementary material [file mmc1.docx]

**Sarah O'Loughlin**

**Managing Editor**

**Data in Brief**

Dear **Sarah O'Loughlin**

We declare that we do not have any interest conflict related with the next manuscript:

Manuscript No.: DIB-D-16-00375

Title: Pharmacological profile of N-(2,6-dichlorophenyl)-2-(4-methyl-1-piperidinyl)acetamide, a novel analogue of lidocaine

Journal Title: Data in Brief

All Authors: Myrna Déciga-Campos

I am signing for my contributors. We work for public universities in México. Funds of this research was supported by each institution and we declare no interest conflict.

Corresponding Author

PhD. Myrna Déciga Campos
